# Hexavalent chromium release over time from a pyrolyzed Cr-bearing tannery sludge

**DOI:** 10.1038/s41598-023-43579-9

**Published:** 2023-09-28

**Authors:** Lisa Ghezzi, Enrico Mugnaioli, Natale Perchiazzi, Celia Duce, Chiara Pelosi, Erika Zamponi, Simone Pollastri, Beatrice Campanella, Massimo Onor, Mahmoud Abdellatief, Fabrizio Franceschini, Riccardo Petrini

**Affiliations:** 1 Department of Earth Science, University of Pisa, Via S. Maria 53, 56126 Pisa, Italy; 2 Department of Chemistry, University of Pisa, via G. Moruzzi 13, 56124 Pisa, Italy; 3 Elettra - Sincrotrone Trieste, in AREA Science Park, Basovizza, 34149 Trieste, Italy; 4 Institute of Chemistry of Organometallic Compounds (ICCOM-CNR) Pisa, Via G. Moruzzi 1, 56124 Pisa, Italy; 5 SESAME Synchrotron, King Hussein Bin Talal St / Box 7, Allan, 19252 Jordan; 6 Environmental Protection Agency of Tuscany (ARPAT), Via Vittorio Veneto, 56127 Pisa, Italy

**Keywords:** Environmental sciences, Chemistry, Materials science

## Abstract

Pyrolysis in an inert atmosphere is a widely applied route to convert tannery wastes into reusable materials. In the present study, the Cr(III) conversion into the toxic hexavalent form in the pyrolyzed tannery waste referred to as KEU was investigated. Ageing experiments and leaching tests demonstrated that the Cr(III)–Cr(VI) inter-conversion occurs in the presence of air at ambient temperature, enhanced by wet environmental conditions. Microstructural analysis revealed that the Cr-primary mineral assemblage formed during pyrolysis (Cr-bearing srebrodolskite and Cr-magnetite spinel) destabilized upon spray water cooling in the last stage of the process. In the evolution from the higher to the lower temperature mineralogy, Cr is incorporated into newly formed CrOOH flakes which likely react in air forming extractable Cr(VI) species. This property transforms KEU from an inert waste to a hazardous material when exposed to ordinary ambient conditions.

## Introduction

Leather manufacturing by tanning processes generates a number of potential environmental threats^1,2^, including the production of substantial quantities of hazardous wastes^3,4^ that require suitable treatments before disposal. Despite the development of novel and cleaner technologies for leather production^5,6^, presently more than 80% of the global leather production involves tanning using chromium salts^7^. Basic Cr(III) sulfate (Cr(OH)SO_4_∙*n*H_2_O) is the primary chromium salt used in the leather industry, because of its high penetration capability into the collagen matrix^8^, which results in a stable fibrous network resistant to bacterial attack and endows skin with excellent hydrothermal stability, elasticity and a comfortable feel^9^. The excess of chromium remains in the tanning liquor and is usually precipitated as trivalent chromium hydroxide using coagulants, which have high removal efficiency^10^. This treatment produces a sludge containing relatively high amounts of Cr, sometimes even exceeding 4 wt%^11,12^.

Various strategies are devised for tannery sludge management and disposal, such as its use in agriculture and landfilling^13,14^. Even if Cr(VI) salts are generally not used in tanning, and Cr in the fresh sludge is expected to persist in its trivalent state due to the high content of organic substances^15^, it is necessary to ensure that Cr(III) remains resistant to the conversion to the hexavalent form during disposal in oxidizing environments^16^, since Cr(VI) compounds are highly toxic and group one human carcinogens^17^. Among the different tannery sludge treatments for re-utilization within a circular economy model^18^, thermal treatments through incineration, gasification and pyrolysis have been widely applied^19–21^. These technologies reduce the waste volume and weight and allow energy recovery^20,22^. During the thermal treatment in oxic or anoxic conditions, wastes undergo a number of reactions and Cr-rich solid phases form, their type mostly depending on sludge composition, temperature of thermal decomposition and heating rate^23–28^. In general, pyrolysis in a controlled reducing atmosphere minimizes the Cr(VI) that may form during the heating treatment and represents a promising and cost-effective technology to treat the sludges, producing a carbon material where Cr is expected to be fixed as non-toxic Cr(III)^29,30^. Pyrolyzed tannery sludge residues free from toxic Cr(VI) can be safely disposed of, converting tannery wastes into a recycling resource^18,31–33^.

The present paper is focused on the characterization of the high Cr-bearing char produced during pyrolysis and sintering of sludge from the tanning district of Santa Croce sull’Arno (Italy), one of the largest in Europe, including more than 250 tanneries. The pyrolysis char, referred to as KEU (Kraftanlagen Energie und Umwelttechnik), has been deemed a model of circular economy. The KEU industrial by-product, considered a non-hazardous waste, was recycled to form inert fill for road construction. The aim of the present work was to investigate KEU stability, addressing the conditions under which Cr(III) may oxidize to Cr(VI). The results provide evidence for time-dependent Cr(III) re-oxidation under aerobic conditions, posing potential environmental threats in KEU reuse and storage.

## Materials and methods

### Tannery sludge processing

Samples were collected during 2022 in the tannery sludge treatment plant of Santa Croce sull’Arno (Tuscany, Italy). The plant, with a capacity of about 500.000 m^3^/year of sludge flow, was built in 2001 with the aim of solving the environmental issues related to the excess sludge landfill disposal. The physical and chemical processes that transform sludge from tannery facilities into KEU are depicted in Supplementary Fig. S1. After thickening, the tannery sludge is first dehydrated by centrifugation and the residue is dried by air blowers at 250 °C to obtain about 90% of total solid content. The solid is pyrolyzed at 900 °C and sintered at 1000–1100 °C by methane/oxygen burners, adopting stoichiometric oxygen/methane ratio to prevent oxidation reactions. Finally, the resulting high-temperature material is quickly cooled by spraying water to produce KEU, a granulate with particle size ranging from about 0.5 to 4 mm (European Waste Code: 19 01 12 mirror non-hazardous).

Despite some fluctuation in the flow rates and in the composition of the incoming sludge, the adoption of a dynamic management system for the wastewater treatment plant for the subsequent pyrolysis ensures the highest chemical and mineralogical reproducibility for KEU. Some heterogeneity in the chemical composition, porosity and crystal size occurs, however repeated sampling indicated that 5 kg of sample guarantee the desired representativeness. About five kilograms of sample were collected by qualified personnel of the Environmental Protection Agency at the end of the whole treatment process (sample: KEU) and from the pyro-sintering section before water cooling (sample: KEU-1). Thus, KEU and KEU-1 samples differ only in having been subjected or not to cooling through water spray nozzles. After collection at the product plant, samples were hermetically stored in vessels at room temperature.

### Chemical characterization, leaching test and Cr(VI) measurements

The chemical composition of sample KEU and KEU-1 was determined by both X-ray fluorescence (XRF) and microwave-assisted acid digestion (EPA method 3051A: 2007) followed by ICP-MS analysis (EPA method 6010 D: 2018). The total Cr and Cr(VI) contents were measured in accordance with standard methods EPA 3051A–EPA 6010D and EN 15192: 2007 respectively. The total organic carbon (TOC) was determined by method EN 15936: 2022. Leaching tests were performed according to the standard EN 12457-2 (compliance test for leaching of granular waste materials and sludges).

The amount of Cr(VI) in aged KEU and KEU-1 samples was selectively extracted by using 0.1 M Na_2_CO_3_^34^. This procedure ensures that no change occurs in the original oxidation state of chromium and allows the determination of Cr(VI) at low concentration^34^. To verify the reproducibility of this method, extraction was repeated twice on different days. Analyses were reproducible within ± 8%. A Dionex DX-500 ion chromatography system with an AD-20 UV–Vis absorbance detector was used for Cr(VI) determination in the extracts. A Hamilton PRP-X-100 analytical column (4 × 250 mm, 5 µm particle size) was used for separation. After elution, a Cr(VI)-complex was formed by post-column derivatization reaction with DPC through a 750 µl knitted reaction coil. Detection limit was 0.2 µg/l (corresponding to 0.1 mg/kg in the solid sample).

### Ageing experiments

The KEU sample was aged in different conditions all open to atmosphere and in the dark: condition A = 25 °C relative humidity (RH) 40%; condition B: 25 °C, RH = 70%; condition C: 25 °C, RH = 90%; condition D: 70 °C (in oven). The KEU-1 sample was aged only in condition C. The 70% and 90% relative humidity conditions were achieved by using a saturated solution of KI or a saturated solution of KCl, while the 40% relative humidity was achieved by storage in a room with controlled humidity. Samples were collected and analyzed for Cr(VI) content every 30 days.

### TGA analysis

Thermogravimetry was performed using a Thermobalance (Q5000IR) equipped with FTIR (Agilent Technologies Cary 640) for Evolved Gas Analysis (EGA). About 50 mg of the samples were heated in Pt crucibles from 25 to 900 °C under nitrogen flow (10 ml/min with 10 °C/min heating rate in case of TGA, and 30 ml/min at 20 °C/min in case of TG-FTIR). Mass calibration was performed using certified mass standards, in the 0–100 mg range, supplied by TA Instruments. The temperature calibration was based on the Curie Point of paramagnetic metals. TG-FTIR spectra of the gas evolved during the thermal scan were acquired every 30 s in the range 600–4000 cm^−1^ with a 4 cm^−1^ width slit. The optical bench was purged with nitrogen to reduce the background signals of water and carbon dioxide present in the atmosphere. A background spectrum was recorded just before each analysis. The TGA spectra were analyzed with the software TA Universal Analysis 2000 (version 4.5A). The FTIR spectra of the evolved gas were analyzed using the software ResPro Evolution (version 5.2.0).

## Microstructural analyses

### X-ray microtomography

The three-dimensional (3D) characterization of the samples was performed using a Bruker SKYSCAN 1174 X-ray computed microtomography scanner operating in cone beam geometry. The X-ray source was a sealed air-cooled X-ray tube operating in a voltage range of 20–50 kV. The used detector was a 1304 × 1024 pixels digital X-ray camera coupled to a P43 scintillator screen. The spatial resolution was set at 6.2 μm/pixel, yielding a maximum field of view of ~ 51.32 mm^2^. The experiments were performed using a 0.25 mm aluminum filter. For each experiment an exposure time of 9.5 s/projection at angle step of 1° over a total scan angle of 180° was set, averaging 4 frames. Reconstruction of the images was performed by the NRecon® server. Three-dimensional renderings were elaborated using the CT-Vox and CT-An software.

### FEG-SEM analysis

Backscattered scanning electron microscopy (SEM) was performed with a FEI Quanta 450 ESEM FEG, equipped with a Schottky FEG source and coupled with an energy-dispersive microanalytic system (EDS) Bruker QUANTAX XFlash Detector 6–10. This apparatus allows acquiring high-resolution imaging (morphological and compositional) of both conductive and non-conductive specimens at nanometer-scale resolution. Acceleration voltage was adjusted in a range between 5 and 20 kV. Samples were deposited dry on a stub covered by a conductive carbon tape.

### XRPD analysis

Synchrotron X-ray powder data (XRPD) in the range 3°–63° 2θ were collected at MS beamline (ID09^35^) at SESAME light source (Allan, Jordan) using a wavelength of 0.82746 Å (15 keV). Powders were loaded into borosilicate glass capillaries and mounted on a standard goniometer head fixed on a spinner. The XRPD experiments were performed in transmission mode (Debye–Scherrer geometry) using a 2-circle diffractometer at room temperature.

A Pilatus 300 K detector with a 172-pixel size was used to collect the diffracted intensities with long exposure time per frame, in the range 30–60 min, to ensure high quality data. The identification of the crystalline phases in the XRPD patterns was carried out through the EVA-Bruker software (Bruker AXS, Karlsruhe, Germany), using the PDF2-2023 database, and furtherly validated through a Rietveld refinement of the XRPD patterns^36^, carried out using the Topas-Academic v7 software^37^.

### XAS analysis

X-ray absorption spectroscopy measurements were performed at the 10.1 X-ray Fluorescence beamline of Elettra synchrotron (Trieste, Italy). Experiments were conducted using a Si(111) monochromator, with standard 45°/45° geometry for both transmission and fluorescence mode measurements, using an Hamamatsu Si-photodiode S3590-09, 10 × 10 mm^2^, 300 µm thickness and an XFlash 5030 SDD detector (Bruker, Berlin, Germany), respectively. Both samples and reference compounds were pressed into 7 mm diameter pellets, containing the element of interest mixed with polyvinylpyrrolidone binder, then sandwiched between two Kapton foils and mounted on a Teflon sample holder. This setup was necessary both to secure the samples, because of the potential hazard represented by Cr(VI) compounds, and to have a system compatible with the working conditions of the Ultra High Vacuum Chamber (UHVC, 10^–7^ mbar) available at the beamline. The monochromator was calibrated in energy prior to XANES measurements using a Cr metal foil.

XANES spectra were collected at room temperature using 3 s per step and a variable energy step as a function of the energy: a large step (5 eV) in the first 200 eV of the spectrum, a smaller step (0.2 eV) in the near-edge region and a k-constant step of 0.03 Å^−1^ further above the absorption edge. Multiple spectra were collected and merged in order to increase the signal-to-noise ratio, when needed. The oxidation state was determined using least-squares Linear Combination Fitting (LCF) based on reference spectra of Cr-bearing compounds with known oxidation state. Background removal, normalization of XANES spectra and LCF analyses (conducted in the energy range − 20 to 30 eV with respect to the absorption edge) were performed using the Athena software package^38^.

### HR-TEM analysis

Samples were gently crushed between two clean glass surfaces, loaded on carbon-coated cupper grids and then mounted on a JEOL double-tilt analytical holder. Transmission electron microscopy (TEM), dark-field scanning-transmission electron microscopy (DF-STEM), energy-dispersive X-ray spectroscopy (EDS) and electron diffraction (ED) experiments were performed with a JEOL JEM-F2000 Multipurpose, working at 200 kV and equipped with Schottky-FEG source. TEM images were acquired with a GATAN RIO16 CMOS camera. EDS point analyses and elemental maps were performed with a JEOL SDD detector and quantified by the JEOL software. ED data were acquired with an ASI Chetaah hybrid-pixel detector on grains rich in chromium. Polycrystalline ring-like ED patterns were acquired from aggregates of particles of few tens of nanometers and analyzed by ImageJ software. Three-dimensional electron diffraction (3DED)^39^ data were collected from single crystals of few hundreds of nanometers in steps of 1° within a total tilt range of about 30°. 3DED data sets were analyzed by PETS2 software for cell parameter determination.

## Results

### Physico-chemical characterization

KEU and KEU-1 samples are chemically indistinguishable; the average and standard deviation of the chemical composition of KEU is reported in Table 1. The main components are total organic carbon (TOC, 18 wt%), iron (16 wt%), calcium (10 wt%), sulphur (5 wt%) and total Cr (2.6 wt%). Hexavalent chromium is below the limit of detection (LOD) suggesting that almost all chromium is in the trivalent form. The averaged results on eluates from the KEU sample leaching show that both physico-chemical parameters and elemental concentration are below the threshold for the disposal of non-hazardous waste material imposed by Italian Regulations, except for chloride and sulphate. It is worth to note that leachates are highly alkaline (pH in the range 9–12).

**Table 1:** Average chemical composition of KEU (wt%).

Elements	Average	SD	n
Fe	16.4	6.1	8
Ca	10.5	2.4	5
S	5.2	1.8	5
Cr	2.6	0.6	8
Na	2.2	0.8	3
Si	2.1	0.3	3
Cl	1.0	0.4	3
P	0.9	0.3	5
Mg	0.8	0.4	3
Al	0.7	0.2	5
Ti	0.5	0.4	5
K	0.4	0.1	5
Zn	0.08	0.02	3
Mn	0.07	0.02	8
Zr	0.05	0.04	6
Cu	0.05	0.06	8
Ba	0.04	0.01	5
Ni	0.03	0.03	6
V	0.02	0.01	7
Sr	0.02	0.002	3
Sb	0.01	0.0006	8
Cr(VI)*	< 0.25	–	5
TOC	18.0	1.0	3

SD standard deviation, n number of samples analyzed.

*Value in mg/kg.

### Ageing experiments

**Figure 1:**
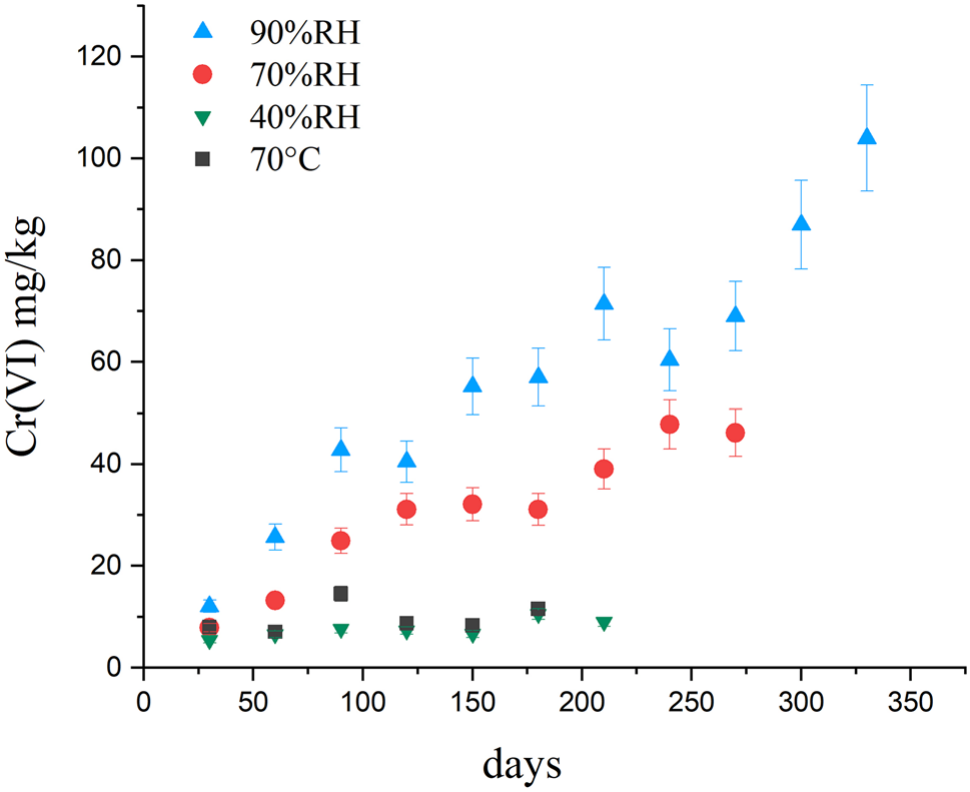
Concentration of Cr(VI) that forms through time in the KEU sample aged in different conditions (see legend on the picture, RH relative humidity).

As previously stated, the Cr(VI) content in the KEU sample was below the detection limit. However, a linear increase in the Cr(VI) content as a function of time was observed by exposing KEU to air in the dark at ambient temperature (25 °C) and under different relative humidity conditions (Fig. 1). In particular, Cr(VI) production was strongly promoted by increasing the relative humidity, reaching 104 mg/kg after 330 days at RH = 90%, while the effect of temperature (up to 70 °C) was negligible. It has to be noted that Cr(VI) does not form when KEU is kept stored in a gas-sealed container at ambient temperature. These observations indicate that the Cr(III) to Cr(VI) conversion occurs within the KEU matrix when exposed to air, and that oxidizing reactions are favoured in wet environments.

Ageing experiments on KEU-1 indicated that Cr(III) is not prone to react in air and wet conditions to form Cr(VI), differently from what observed for the KEU sample. Indeed, the concentration of Cr(VI) in KEU-1 slightly increased from below the limit of detection (0.1 mg/kg) right after its production, to 2.5 mg/kg after 180 days.

### TGA analysis

**Figure 2:**
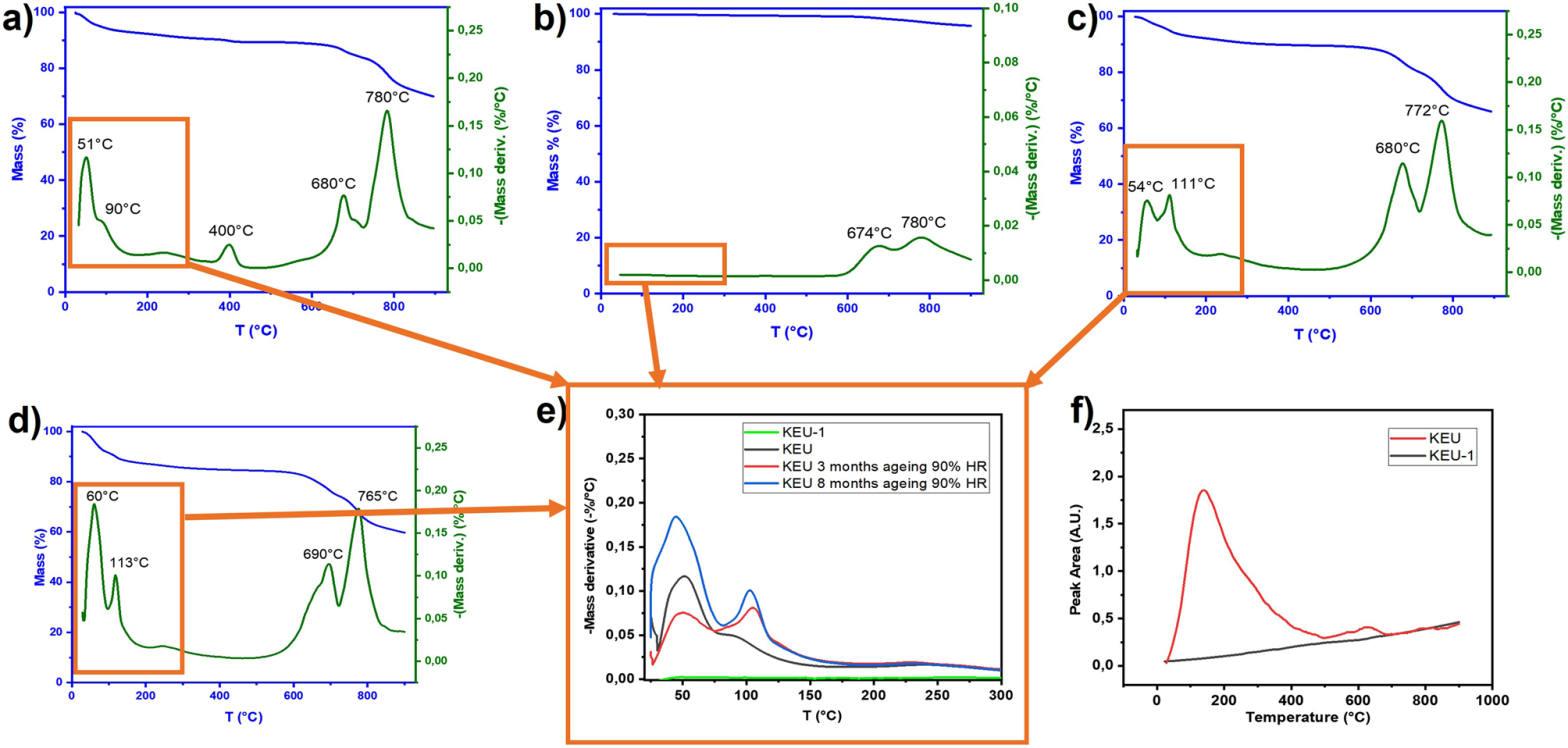
Thermogravimetric curves and their derivative for KEU (**a**), KEU-1 (**b**) and KEU after 3 (**c**) and 8 (**d**) months of ageing at 90% RH. (**e**) Zoom on the DTG profiles of the samples described in the text. (**f**) Profile of water evolved during the thermal degradation process for KEU and KEU-1.

TGA analysis shows that heating KEU under nitrogen atmosphere from 25 to 900 °C leads to a total mass loss of about 30% (Fig. 2a). The analysis of the gases evolved during the thermal degradation by FTIR spectroscopy (Supplementary Fig. S2) permitted to assign the first degradation step in the temperature range up to 180 °C to the loss of H_2_O (7%). The second step from about 200 to 500 °C was attributed to the simultaneous loss of H_2_O and CO_2_ (4%), the third (at about 680 °C with a mass loss of 6%) and fourth step (at about 780 °C with a mass loss of 14%) to the simultaneous loss of CO_2_, CO, and water. The CO and CO_2_ release in this temperature range might be attributed to both iron oxide reduction by elemental carbon^40^ and thermal decomposition of calcium carbonate. On the other hand, the thermal degradation profile obtained for KEU-1 shows only a mass loss of ca 4% with two maxima observed in DTG at about 680 °C and 780 °C (ascribable to the loss of CO, CO_2_ and water), without any water loss at lower temperature (Fig. 2b, Supplementary Fig. S3). To highlight the different water release from KEU and KEU-1, the profile of water evolved with the temperature from the two samples, obtained by plotting the peak area of the H–O–H bending band at 1700–1500 cm^−1^^41^, is reported in Fig. 2f.

The thermogravimetric analysis was also performed on the KEU sample aged for 3 and 8 months at RH 90% (Fig. 2c,d). A close inspection of the DTG profiles below 300 °C (Fig. 2e) highlights the presence of a first peak (centred at around 60 °C), related to the release of moisture, and a second peak (centred at almost 110 °C) related to the loss of more strongly bonded water. Both peaks are absent in KEU-1, and increase in KEU over time, reflecting the entry of aqueous vapours in KEU pores.

## Microstructural characterization

### X-ray microtomography

The KEU sample has a heterogeneous tridimensional framework, composed of spots with high density surrounded by a lower density matrix. The image reconstruction shows the presence of irregular micrometric pores, often elongated, with a width of ca 100 µm and lengths which reach 500 µm.

**Figure 3:**
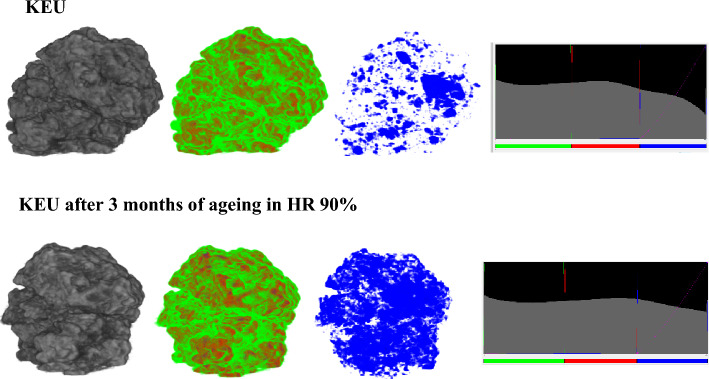
Morphology of the sample at time 0 and after 3 months of accelerate ageing at 90% of HR. The grey image highlights the material density, in increasing scale from white to black. The coloured images highlight the low, medium, and high density respectively in green, red and blue. The pixel profile is reported on the right.

The same analysis performed on aged KEU samples (Fig. 3) shows that ageing at 90% relative humidity induces an increase of the high-density areas due to water infiltration in the pores of the material.

### FEG-SEM analysis

**Figure 4:**
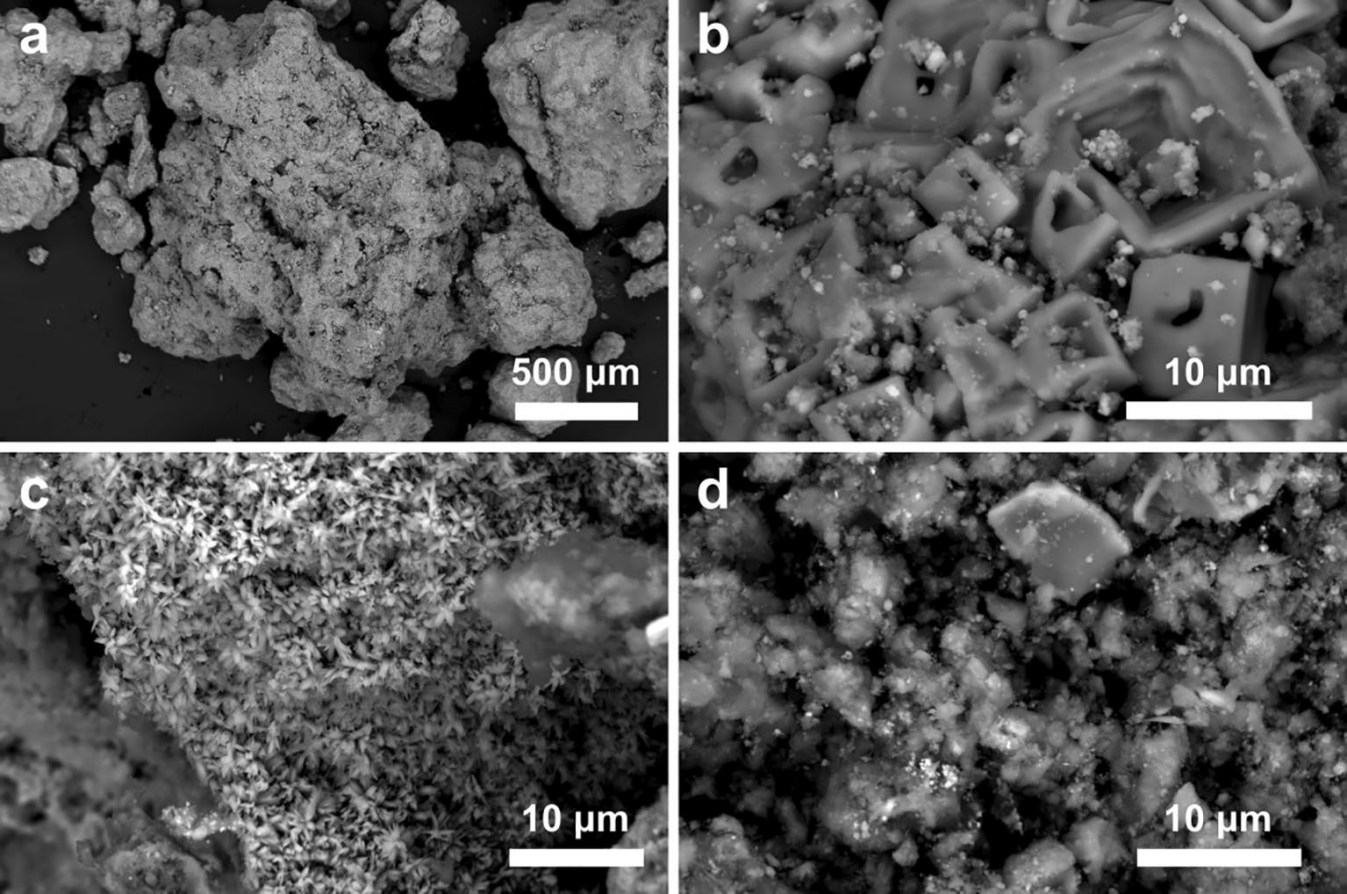
SEM images of KEU sample. (**a**) Typical aspect, made of a carbonaceous amorphous substrate and different crystalline phases. (**b**) Area occupied by concave inter-growth of micrometric Na chloride crystals and by smaller aggregates of prismatic Fe oxides/hydroxides. (**c**) Area occupied by Fe oxide/hydroxides with typical radial growth. (**d**) Area occupied by a complex association of Fe oxides/hydroxides, Ca carbonate, Ca sulphate and silicates. The bright spots in the lower-left part of the image are nanometric particles rich in Cr.

The KEU sample appears as aggregated pellets of few millimeters or smaller size, mostly made of fine black dust and subordinately by reddish or bright sub-millimetric spots. FEG-SEM analysis shows that the sample is extremely heterogeneous. KEU pellets are mostly made by a fine, amorphous and porous carbonaceous matrix (Fig. 4a). Locally, areas occupied by several crystalline phases are observed. Crystals are up to few micrometers in size and generally have a well-developed euhedral habit (Fig. 4b). The most abundant crystalline phases are Ca carbonates, Ca sulphates and Fe oxides/hydroxides. The latter have a planar or prismatic habit, typically with radial growth (Fig. 4c). Other common crystalline phases are Na chloride, Ti oxides and silicates (mostly quartz). Cr shows an extremely variable concentration from point to point and does not show any significant correlation with other major elements (Ca, Fe, S, Na, Cl, Si, Ti). The size of most crystalline particles, and in particular the Cr-rich particles, is too small for quantitative EDS analysis by SEM. Cr is particularly abundant in aggregates of sub-micrometric rounded particles with high density (i.e. shining in back-scattered electron images, Fig. 4d). Conversely, large crystals containing Ca and Fe do not show significant Cr concentration.

### XRPD measurements

The results of the Rietveld refinement are reported in Table 2 and the XRPD patterns in the 2θ range 43°–45° of the KEU, KEU-1 and aged KEU samples, together with their assignments, are shown in Supplementary Fig. S4. The presence of a relevant amount of an amorphous component in KEU and, to a lesser extent, in aged KEU is suggested by the presence of a low angle bump in the XRPD patterns.

**Table 2:** Mineralogy and modal abundancies (wt%) of the crystalline phases resulting from the Rietveld refinement of samples KEU-1, KEU and aged KEU.

Mineral phases	KEU-1 (wt%)	KEU (wt%)	Aged KEU (wt%)
Srebrodolskite Ca_2_Fe_2_O_5_	38	–	–
Magnetite Fe_3_O_4_	36	17	19
Calcite CaCO_3_	11	8	11
CaFe_3_O_5_	8	–	–
Wustite FeO	7	6	6
Bassanite CaSO_4_·0.5 H_2_O	–	18	15
Vaterite CaCO_3_	–	18	12
Hydroxylapatite Ca_5_(PO_4_)_3_(OH)	–	14	16
Aragonite CaCO_3_	–	6	–
Gypsum CaSO_4_·H_2_O	–	–	8
Fe–C alloy	–	6	5
α-Iron	–	5	4
Goethite FeO(OH)	–	3	2
Eskolaite Cr_2_O_3_	–	1	1

The identification of the constituent phases made through the EVA software is confirmed by the Rietveld refinements, and the modal ratios determined for the crystalline phases present can be used for comparing the mineralogy of the three samples.

As it can be seen from Table 2 and Supplementary Fig. S4, the diffraction patterns of KEU and aged KEU show a similar mineralogy, markedly different from the mineralogical composition of KEU-1.

In KEU and aged KEU the major phases are the hydrated phase bassanite and vaterite, magnetite, hydroxyapatite and calcite. The refined cell parameters for magnetite are very close, namely a = 8.390(1) Å for KEU and a = 8.385(1) Å for aged KEU, both consistent with a pure or Cr-bearing magnetite^42^. Approximately the same quantities of wustite, Fe–C alloy, α-iron, goethite and eskolaite are also detected in the two samples, while in aged KEU the disappearance of aragonite and a decrease in bassanite and vaterite content, with an increase of the more stable phases calcite and gypsum, are observed.

With respect to KEU, the KEU-1 sample consists of anhydrous phases, with magnetite and srebrodolskite as essential constituents. The magnetite cell parameter a = 8.382(1) Å, refined through the Rietveld method, is still consistent with a pure or Cr-bearing magnetite^42^. The cell volume of srebrodolskite, refined through the Rietveld method, is slightly contracted (445.7 vs 448.4 Å^3^) with respect to the literature value^43^. Taking into account the bulk chemical composition of the material, this could be attributed to the replacement, in this phase, of Fe^3+^ with Cr^3+^ (R_i_(Fe^3+^) = 0.645 Å, R_i_(Cr^3+^) 0.615 Å.

### XAS measurements

**Figure 5:**
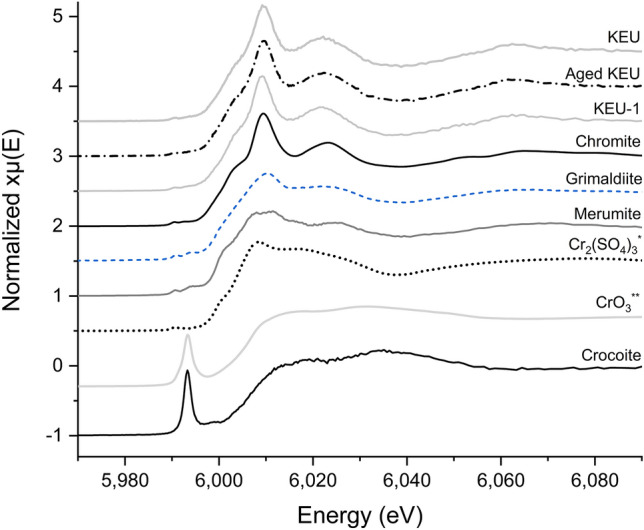
Normalized XANES spectra of samples (KEU, aged KEU and KEU-1) and Cr-bearing reference compounds (sorted from Cr(III) to Cr(VI), from the top to the bottom) collected at the Cr K-edge. As can be seen, spectra of samples are almost indistinguishable, apart of a minor red shift of the white line of KEU-1 spectrum. On the contrary, different features and energy shifts can be appreciated for the spectra from standard compounds, in particular the typical sharp pre-edge peak for Cr(VI) at about 5993 eV. *Reference^44^. **From the beamline database.

The XANES spectra of KEU, KEU-1 and aged KEU samples and of reference compounds (Cr_2_(SO_4_)_3_)^44^, are shown in Fig. 5. It is observed that the main features of the sample spectra resemble those of the chromite reference compound. This is confirmed by the LCF results, indicating that in all samples an important fraction of Cr (varying from roughly 80 to 60%) belongs to Cr-spinel, i.e. Cr-rich magnetite.

However, from XAS results it is possible to state that the majority of Cr has a similar chemical environment in the different samples and consequently, any difference in Cr oxidation/release between the samples must be related to a small Cr fraction with respect to the total Cr content. According to the pre-edge characteristic peak of Cr(VI) at about 5993.0 eV (Fig. 5), there is no detectable evidence of Cr(VI) species in any of the samples, not even in aged KEU where Cr(VI) is expected (XAS detection limit around 1%).

### HR-TEM analysis

**Figure 6:**
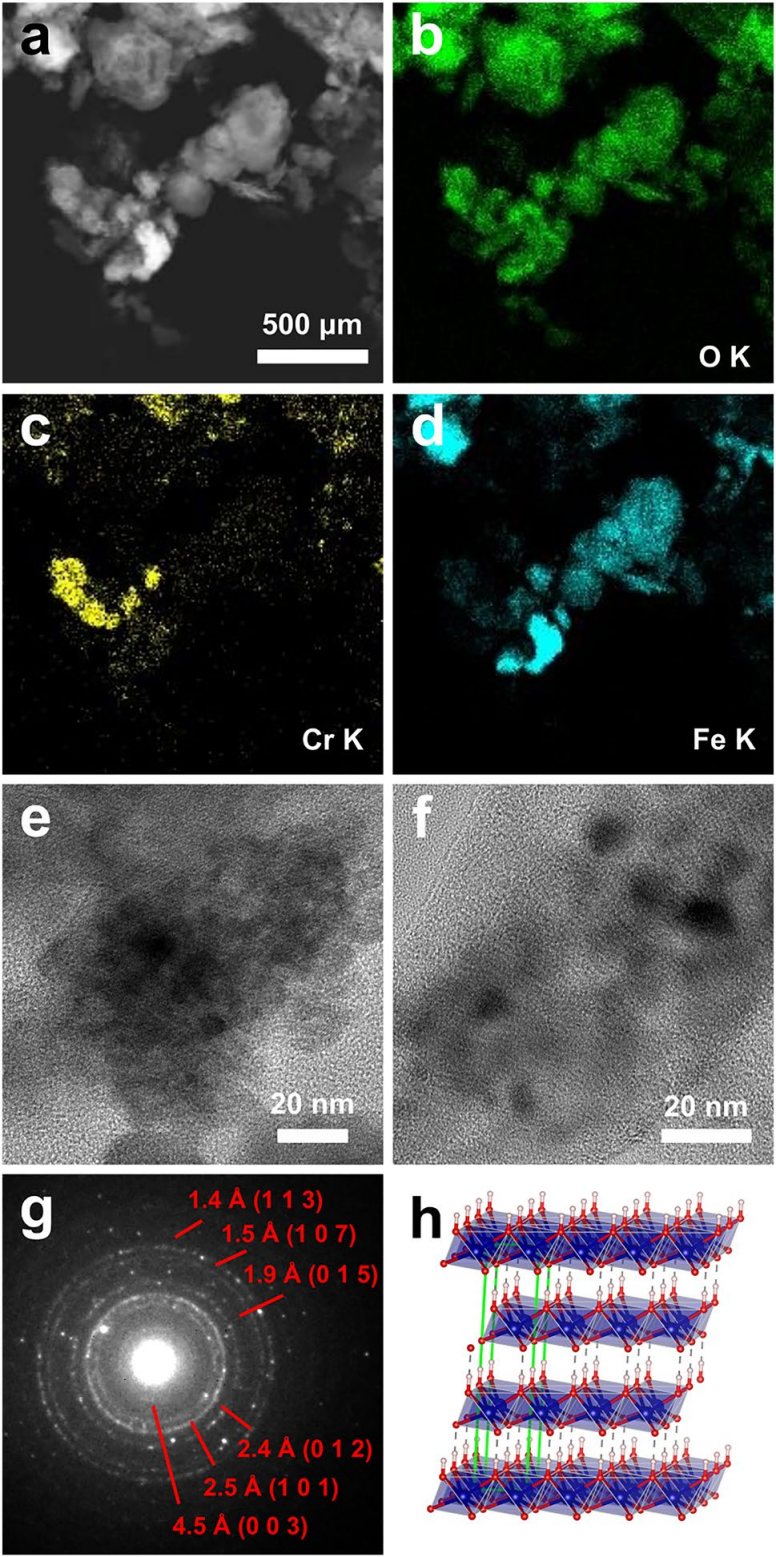
TEM analysis of KEU sample. (**a**) Dark-field STEM image of an aggregate of particles and related EDS map for (**b**) O_K line, (**c**) Cr_K line and (d) Fe_K line. Cr signal concentrates in rounded flaky aggregates and correlate with O signal but not with Fe signal. (**e,f**) Bright-field TEM images showing that the aggregates containing Cr are made of flakes and rounded particles of about 5–10 nm in diameter. (**g**) Polycrystalline ring-like electron diffraction pattern obtained from a Cr rich aggregate. Rings are indexed according with the strongest peaks of *α*-CrOOH (grimaldiite). (**h**) The atomic structure of grimaldiite, where CrOOH layers are kept together by relatively weak hydrogen bonds. Cell edges are sketched in green, Cr atoms in blue, O atoms in red and H atoms in grey.

TEM analysis confirmed that the KEU sample is extremely heterogeneous. Several aggregates of particles, generally few micrometers in size, were first analyzed by STEM dark-field imaging and EDS mapping. Fe oxides/hydroxides, Ca carbonates, Ca sulphates, Na chloride, Ca phosphates, silicates (among which quartz, phyllosilicates and feldspars), rare Ti oxides, rare Mg carbonates and porous aggregates rich in Cr were identified (Fig. 6, Fig. S5).

Notably, Fe oxides/hydroxides appear as particles with a wide size range, from few tens to hundreds of nanometers. The largest particles have a massive habit with sharp edges. EDS point analyses revealed that these particles generally do not contain measurable amounts of Cr or any other element different from Fe and O (H cannot be measured) (Fig. S6a).

The data indicate that Cr is mostly concentrated in flaky aggregates, which also contain O as a main constituent (Fig. 6a–d). Generally, such aggregates include variable and subordinate amounts of other elements, among which Fe, Ca, S, Na and Cl (Fig. S6b). The Cr-rich aggregates have a porous and irregular shape. Inside, it is possible to recognize both flakes and crystalline nanoparticles of about 5–10 nm in diameter (Fig. 6e,f). Due to the strong aggregation of flakes and particles, it was not possible to identify the exact source of Cr. However, the Cr-rich aggregates produced a diffused ring-like polycrystalline electron diffraction pattern (Fig. 6g) which is consistent with the strongest reflections of the rhombohedral cell of *α*-CrOOH (mineral grimaldiite). The alleged ring (003) appears relatively weak, consistently with the likely disorder associated with the structural stacking of COOH layers (Fig. 6h).

The KEU-1 sample also appeared heterogeneous. Compared with KEU, large particles with size of few hundreds of nanometers were relatively more common. The most abundant class of large particles was mostly made of Fe and O, but could contain significant amounts of Mg, Al and Cr. EDS spot analyses revealed that the amount or Cr varies from particle to particle, with an average Cr:Fe ratio of about 1:10 (Figs. S6c, S7). 3DED data revealed that these particles are generally single-crystal, with a *F*-centered cubic cell and *a* = 8.4(2) Å (Fig. S8), consistent with the cells of magnetite (Fe^2+^Fe^3+^_2_O_4_) or maghemite (Fe^3+^_2.67_O_4_). A second class of large particles, relatively less common, are mostly made of Ca, Fe and O and contain significant amounts of Al and Cr (Fig. S6d). 3DED data revealed that these particles are also single-crystal, with a primitive orthorhombic cell and *a* = 5.4(1) Å, *b* = 14.9(3) Å and *c* = 5.7(1) Å (Fig. S9), consistent with the cell of srebrodolskite (ideal formula Ca_2_Fe^3+^_2_O_5_). Due to the relative abundance and the large particle size, magnetite/maghemite and srebrodolskite particles likely host most of the overall Cr detected in the sample. Still, it was possible to spot rare nanometric particles substantially made of Cr and O only (Fig. S10).

Other phases detected in these samples include silicates, Ti oxides and mixed Si-Ca oxides. Halos and particles containing Ca, S, Na, Cl, P and Mg were also present in the samples, but their accurate characterization is not trivial due to the small grain size and the poor crystallinity.

## Discussion

The thermal treatment under absent or limited oxygen conditions is a promising technique for the management of Cr-tannery sludge, producing a carbon material (KEU) that reduces the problem of toxicity and converts a waste into a value-added product. However, the environmental safety of pyrolyzed tannery wastes over time requires to be carefully evaluated before being used as a fill material. Two types of KEU samples were analyzed, i.e. KEU-1 and KEU, collected before and after the final water cooling step during tannery sludge pyrosintering process, respectively. The two materials, although having identical chemical composition, show significant differences in mineralogy. In particular, mineralogical data suggest that chromium in KEU-1 is hosted in its trivalent state in the Cr-bearing mineral phases Cr-srebrodolskite and Cr-spinel (Cr-rich magnetite), while in KEU trivalent chromium is hosted in Cr-spinel (Cr-rich magnetite) and grimaldiite.

Chromium spinel in nature is recognized as a high-temperature mineral in magmatic systems; srebrodolskite is known from very few localities in particular geological settings, e.g. in the pyrometamorphic mineral assemblage^45^, or found as an artificial compound in metallurgical slags^46^. Eventually, srebrodolskite in KEU-1 might have been formed by solid-state CaO–FeO high temperature interactions under low oxygen partial pressure since some iron exists in its trivalent state^47^, incorporating Cr_2_O_3_ partially substituting for Fe_2_O_3_. The mineralogical data on KEU show that srebrodolskite totally disappeared upon cooling. A possible reaction might be the srebrodolskite decomposition by carbonation during hot-to-cold pathway, according to the literature^48^. Indeed, carbonation thermodynamics indicates that the reaction is favored (negative Gibbs free energy of reaction) decreasing the temperature, depending on the CO_2_ partial pressure^48^. The hypothesis of srebrodolskite carbonation could be reasonable considering the carbonates in KEU-1, even if the CO_2_ uptake kinetics requires further investigation. In addition, the amount of magnetite spinel significantly decreased, changing its primary composition and, in particular, loosing chromium as indicated by TEM-EDS analysis. Indeed, Cr-spinel in natural settings is sensitive to chemical modification during hydrothermal alteration^49^. The reaction mechanism involves the increase of the Fe:Cr ratio, providing Cr for secondary phases. These observations indicate that the pristine Cr-hosting mineralogy of KEU-1 is destabilized upon the spray water cooling procedure. In this process, chromium is released and is incorporated into the newly formed Cr-oxyhydroxides CrOOH (grimaldiite) flakes that characterize KEU. Grimaldiite rarely occurs in natural settings; hydrothermal synthesis experiments support the hypothesis that the genesis of natural α-CrOOH is due to medium temperature (340–360 °C) hydrothermal alteration of the primary Cr-bearing minerals^50^. The water cooling process that produces KEU likely mimics such hydrothermal conditions, freezing the equilibrium that causes the synthesis of the grimaldiite flakes.

It is generally assumed that grimaldiite is quite stable against Cr(III) oxidation, even under hydrothermal conditions, at temperatures below about 350 °C. However, oxidation of Cr(III) may result from O_2_ adsorption on the surface of the chromium hydroxide, with the formation of extractable Cr(VI)^51^. Moreover, Liu et al.^52^, on the basis of experimental results and theoretical analysis, reported that Cr(III) in CrOOH has the potential to be oxidized to CrO_4_^2−^ by reacting with chemically adsorbed oxygen in alkaline conditions, and that the oxidation of chromium hydroxide CrOOH to form chromate is thermodynamically feasible even at ambient temperature.

Ageing experiments on KEU clearly demonstrate that the Cr(III)–Cr(VI) inter-conversion occurs over time in the presence of air and moisture, differently from what observed in KEU-1. This indicates that KEU material becomes reactive when disposed in wet environmental conditions, forming Cr(VI). On the basis of the different mineralogy of KEU and KEU-1, this can be attributed to the oxidation of Cr(III) in grimaldiite by oxygen. Indeed, it is known that Cr(III) is readily oxidized by Mn(IV)-oxides^53^; however, the Mn content in KEU is below 0.1 wt%, and XAS Mn K-edge data indicate that the Mn oxidation state is expected to vary between 2^+^ and 3^+^. Hence, a role of Mn oxides in the observed Cr oxidation process is to be ruled out. A significant interaction between other metallic components, such as alkali and alkaline earth metals, and Cr(III)–Cr(VI) species during the ageing experiments at ambient temperature seems also to be excluded, since such reactions are confined to high temperature.

The observed chromium(III) oxidation in the KEU material produced through a pyrolysis process with a final water spraying cooling step renders KEU a hazardous material, which is able to release hexavalent chromium to circulating water when exposed to ordinary ambient conditions. It is worth noting that KEU is highly porous and characterized by a strong tendency to absorb water vapor when exposed to humid air, giving rise to highly alkaline hydrous conditions which trigger grimaldiite oxidation yielding water extractable Cr(VI). It is likely that the oxides of alkali and alkaline earth metals formed during pyrolysis, reacting with water, form hydroxides and produce an alkaline solution.

The behavior of KEU is hence of long-term environmental concern, also considering that about 200,000 tons of KEU have been placed in the environment in Tuscany region (Italy). Monitoring actions must therefore be planned to minimize human exposure, in particular to prevent the contamination of deep groundwater exploited for drinkable water supply. In addition, the process of KEU manufacturing should be improved, including quenching using cooling media different from water and/or improving the spray cooling technology, in order to maintain the pristine crystalline structure of the pyrolyzed tannery waste and to prevent the formation of unwanted Cr-hydroxide constituents. Tests should be carried out to render KEU a safe recycled or reusable material.

## Conclusions

A plant producing a pyrosintered granulate (KEU, European Waste Code: 19 01 12 mirror non-hazardous) was built to manage the excess volume of Cr-bearing sludge produced in the Santa Croce sull’Arno tannery district (Tuscany, Italy), one of the largest in Europe. KEU was widely distributed in the environment, recycled as inert fill material used as a base in the construction of roads. Ageing experiments demonstrate that in KEU a time-dependent Cr(III)–Cr(VI) inter-conversion occurs at ambient temperature in the presence of air, and that the reactivity of the solid is strongly enhanced in wet environmental conditions. Microstructural analysis reveals that the pristine stable Cr(III) high-temperature mineralogy of KEU that forms during pyrosintering is strongly destabilized upon spray water cooling, allowing Cr(III) to be released and incorporated into newly formed α-CrOOH (grimaldiite) flakes. The trivalent chromium in CrOOH oxyhydroxides has the potential to be oxidized to Cr(VI), which become easily leachable by water, making KEU an hazardous material when exposed to ordinary ambient conditions. The water spray cooling in the last stage of the KEU processing plant needs to be further improved to avoid chromium oxyhydroxides to form.

### Supplementary Information

Supplementary Figures.

## Data Availability

All data generated or analyzed during this study are included in this published article and its Supplementary Information files.
