# Supplementary material for: Hexavalent chromium release over time from a pyrolyzed Cr-bearing tannery sludge
Source: Sci Rep. 2023 Sep 28;13:16283. doi: 10.1038/s41598-023-43579-9 (PMC10539330; doi:10.1038/s41598-023-43579-9)
Supplement: Supplementary file 1 — Supplementary Figures. [file 41598_2023_43579_MOESM1_ESM.pdf]

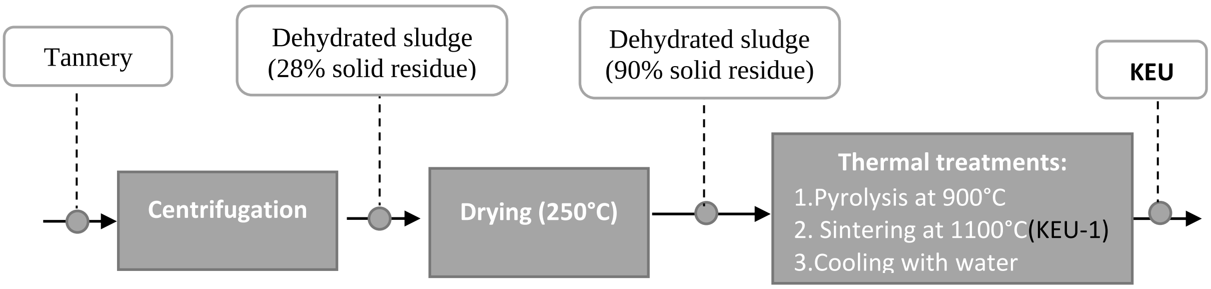

Figure S1. Production scheme for KEU. The position of KEU and KEU-1 samples is indicated.

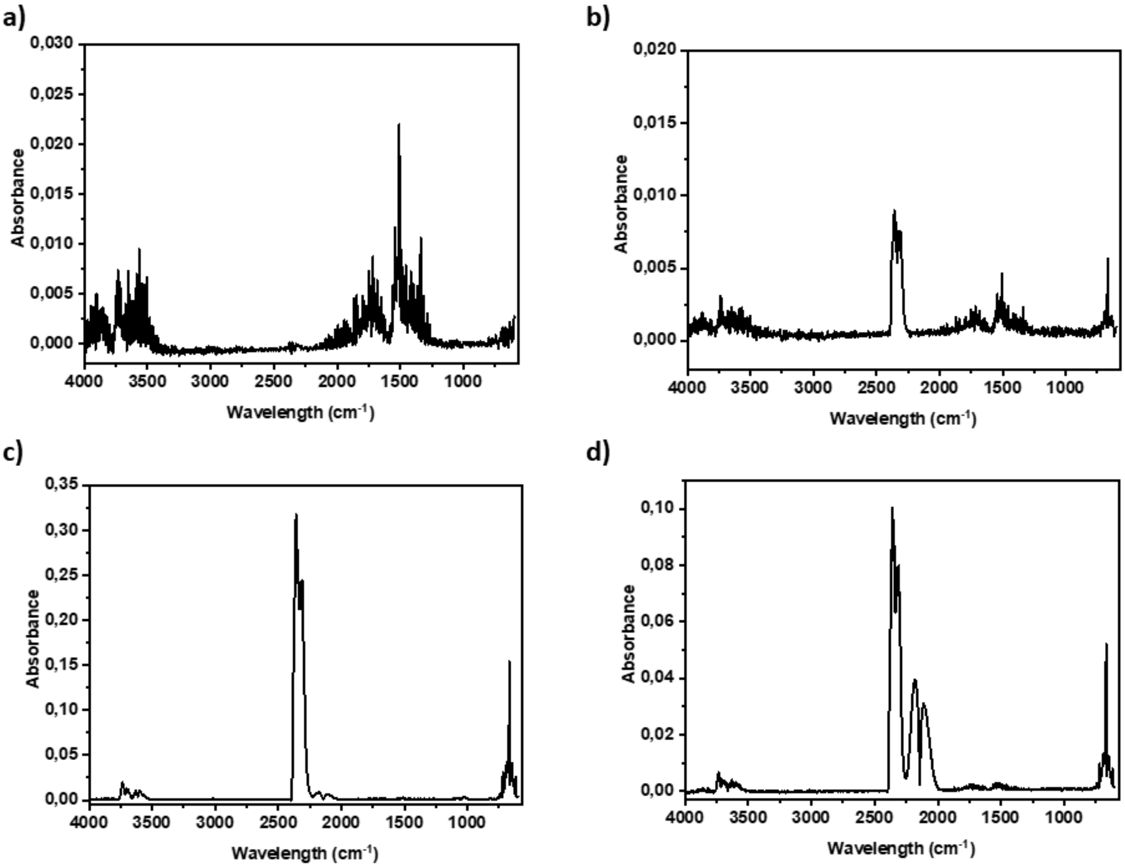

Figure S2. FTIR spectra of gas evolved during thermal degradation of KEU at temperature a) 100°C; b) 400°C; c) 665°C; d) 785°C.

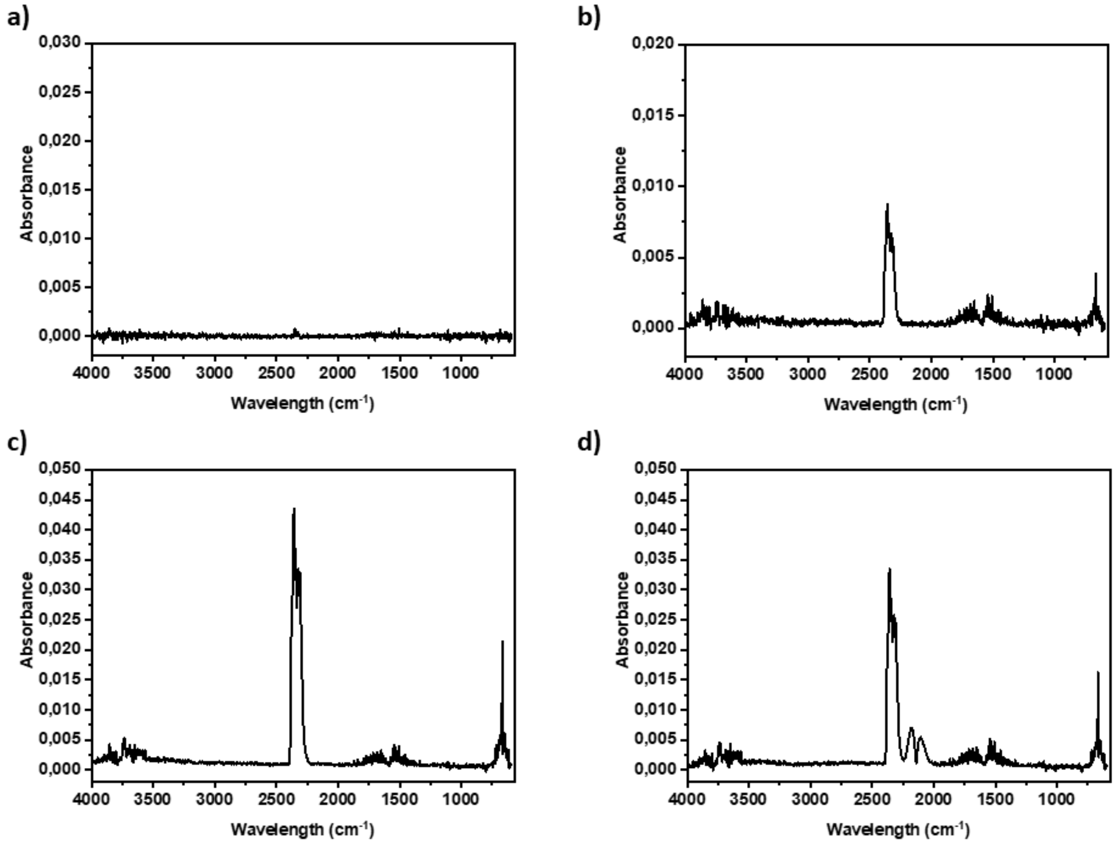

Figure S3. FTIR spectra of gas evolved during thermal degradation of KEU-1 at temperature a) 100°C; b) 400°C; c) 665°C; d) 785°C.

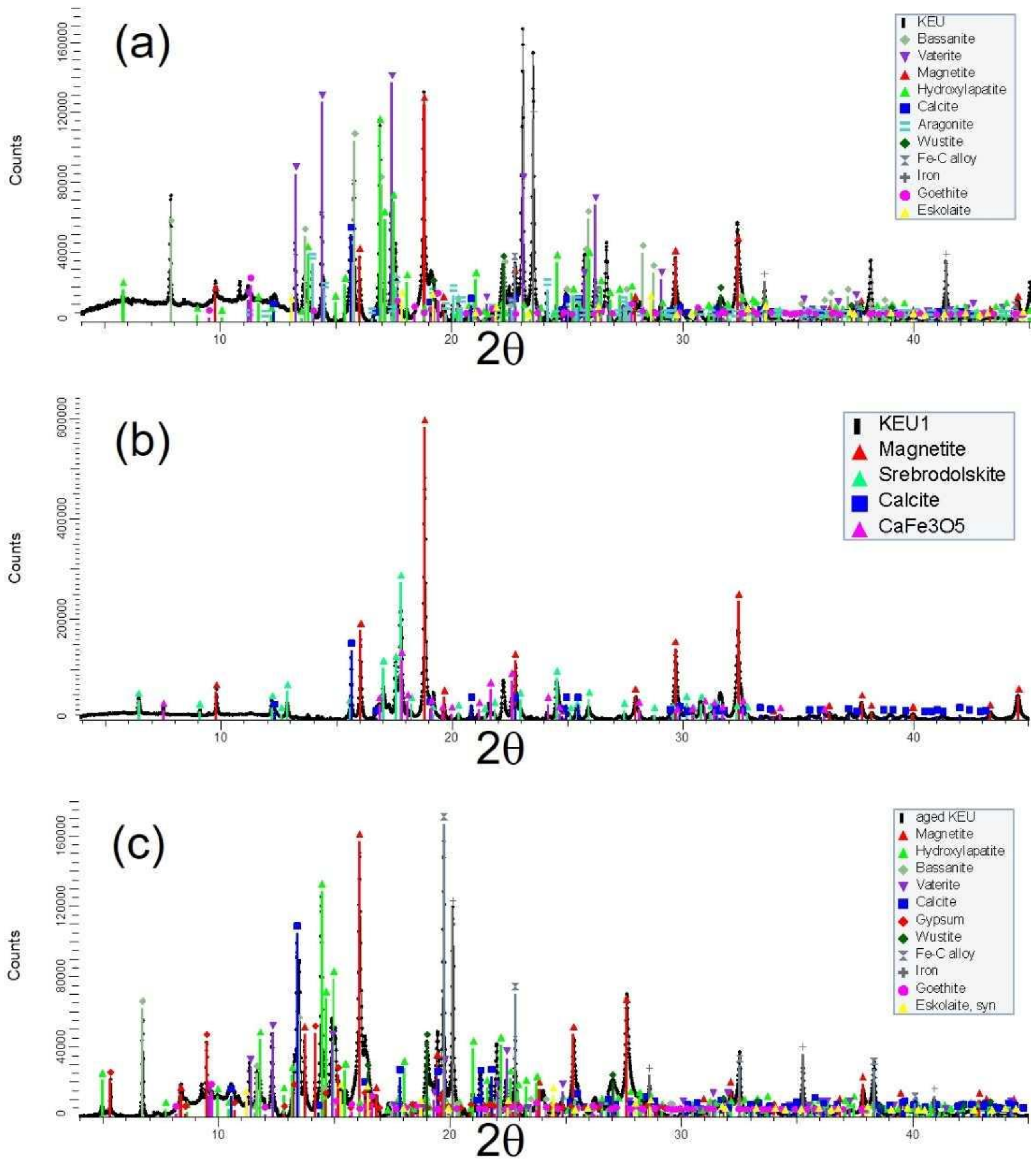

Figure S4. XRPD synchrotron background-subtracted pattern of (a) KEU, (b) KEU-1 and (c) aged KEU (R.H 90%, 210 days) with the identified phases.

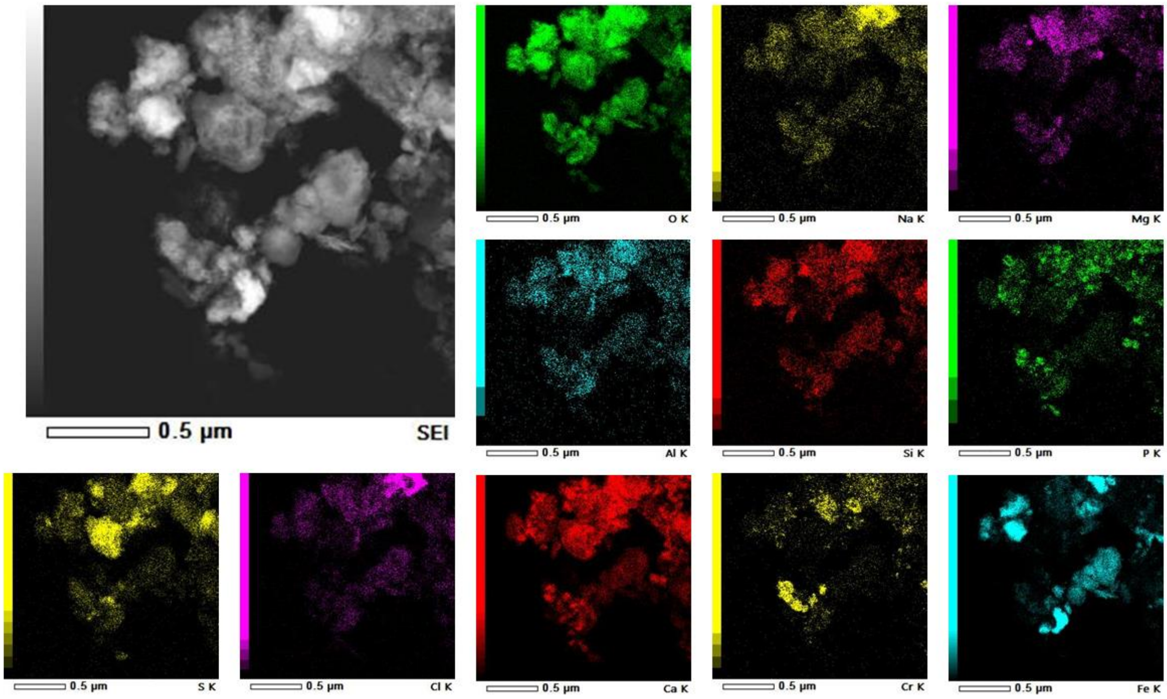

Figure S5 STEM-EDS map of the same aggregate shown in Fig. 6 and collected at the end of keu implant on the conveyor roller. The Cr distribution does not follow any other major element beside O.

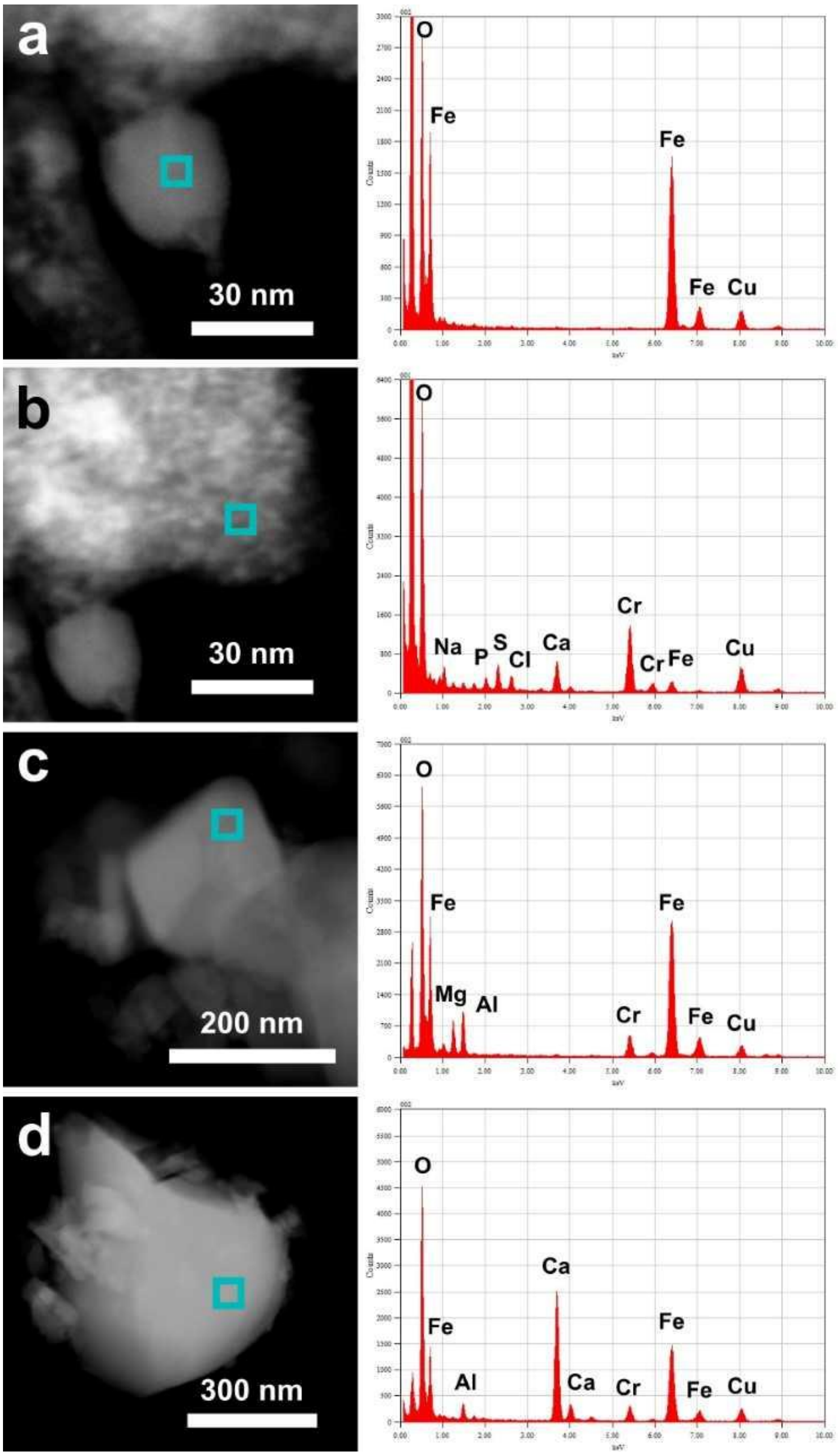

Figure S6. STEM-EDS analysis of KEU and KEU-1 samples. (a) Dark-field STEM and EDS spectrum of an Fe oxide/hydroxide of KEU sample, containing no measurable amount of Cr or other metals. (b) Dark-field STEM and EDS spectrum of a flaky aggregate rich in Cr and O in KEU sample. Other elements are also present, but in quantities variable for different aggregates. In the specific case, the relative high signal of S and Ca may indicate the presence of Ca sulphates. Cr is however always the most abundant element beside O. (c) Dark-field STEM and EDS spectrum of an Fe oxide/hydroxide in KEU-1 sample, showing a significant amount of Al, Mn and Cr. (d) Dark-field STEM and EDS spectrum of a srebrodolskite in KEU-1 sample, showing high amounts of Ca, Fe and O, together with minor amounts of Cr and Al. In all spectra, the Cu signal comes from the TEM grid. The blue square indicates the exact area where EDS data were obtained.

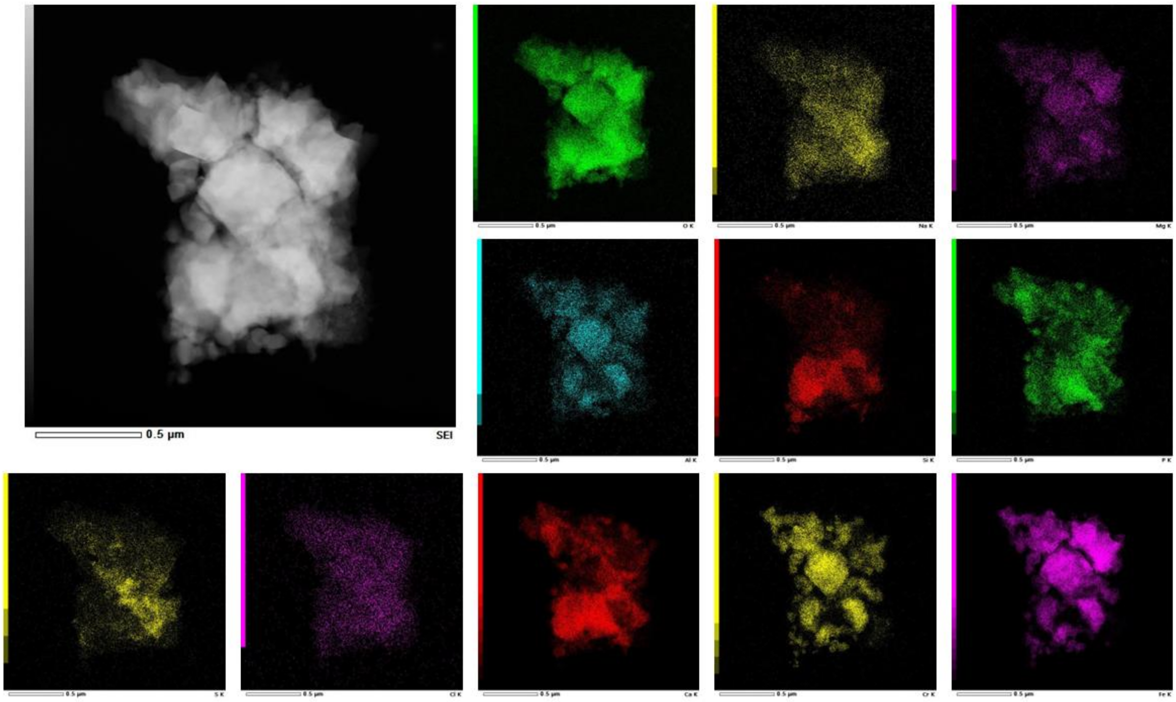

Figure S7. STEM-EDS map of the keu aggregate collected in the pyrolysis furnace. The Cr distribution follows the distribution of Fe, Al, Mg and O, inside particles with composition and cell close to magnetite/maghemite.

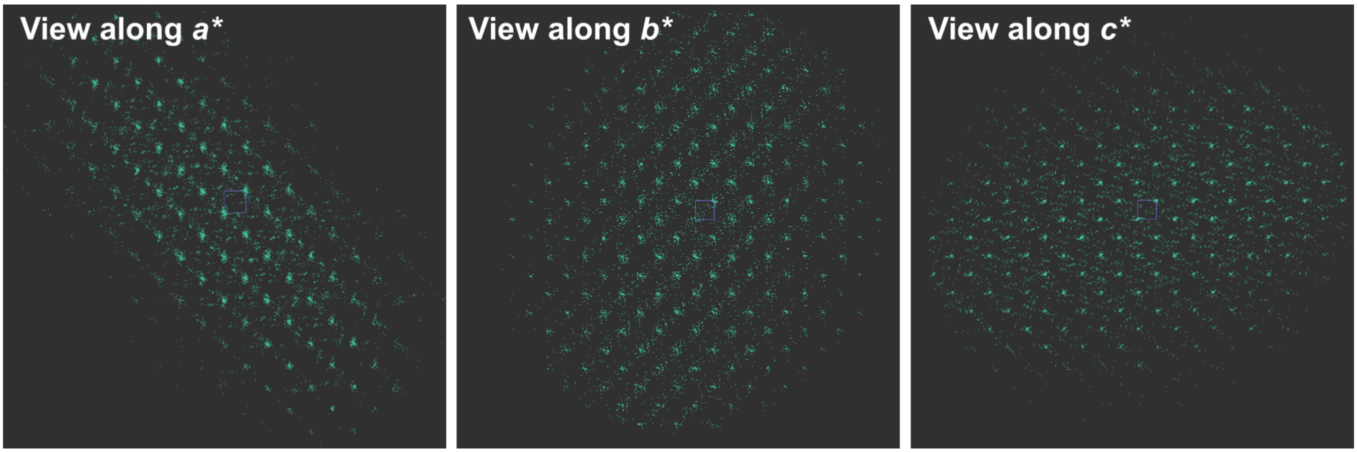

Figure S8. Reconstructed diffraction volume from 3DED data of a Cr-bearing magnetite/maghemite crystal collected in the sintering furnace. The cell, sketched in blue, is *F*-centered cubic with a = 8.4(2) Å.

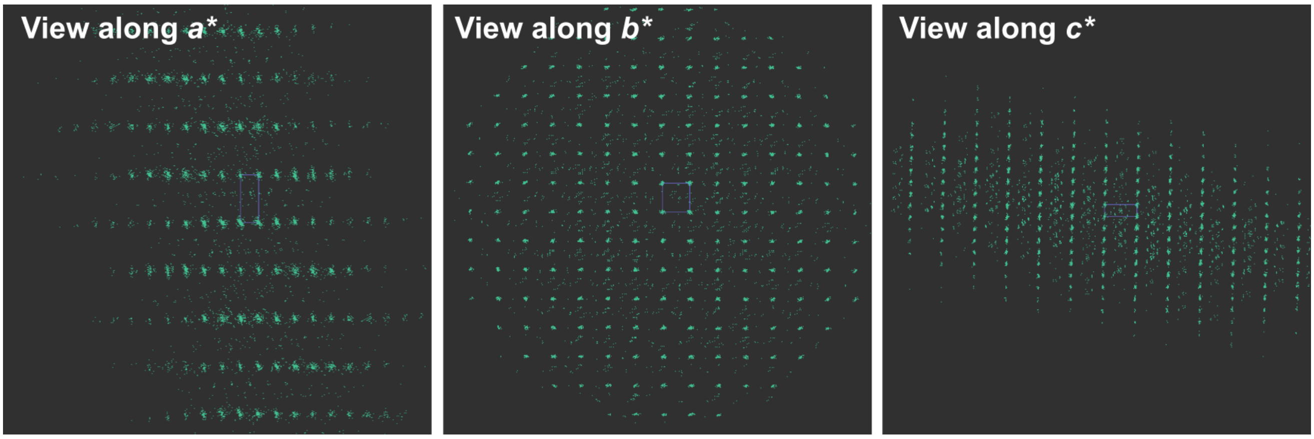

Figure S9. Reconstructed diffraction volume from 3DED data of a Cr-bearing srebrodolskite crystal collected in the pyrolysis furnace. The cell, sketched in blue, is primitive orthrombic with a = 5.4(1) Å, b = 14.9(3) Å and c = 5.7(1) Å.

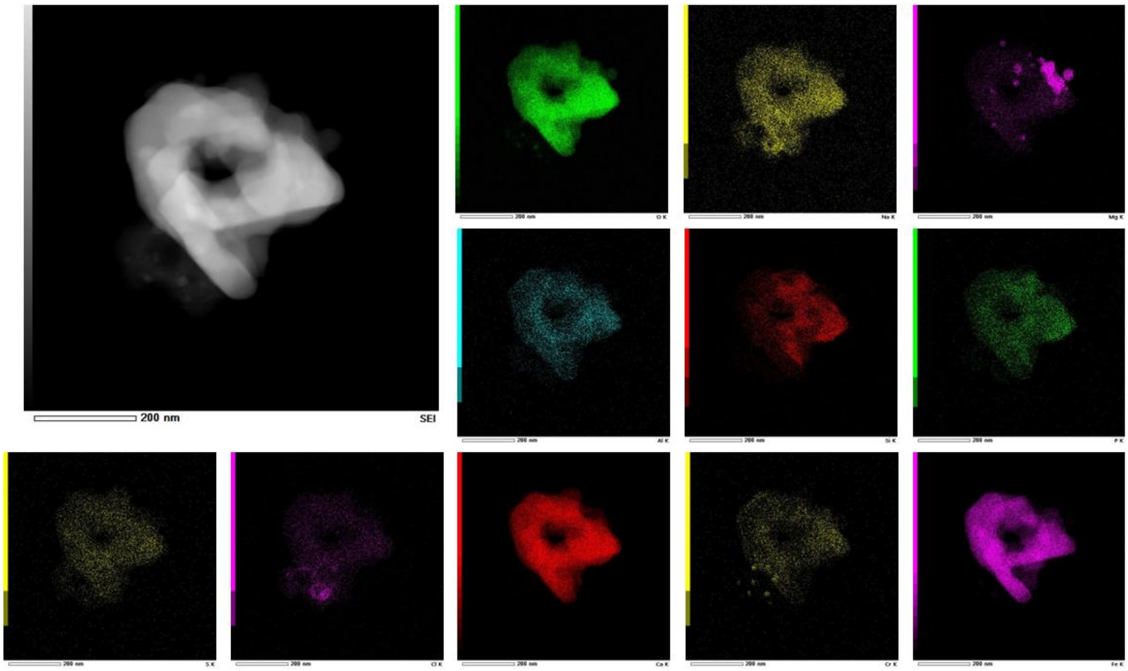

Figure S10. STEM-EDS map of an aggregate of srebrodolskite grains collected in the sintering furnace. Cr mostly follows the distribution of Ca, Fe, and O inside srebrodolskite, but few small nano-particles containing only Cr and O are also visible in the bottom-left part of the map.
